# Biological subtyping of schizophrenia and relationship with clinical features: a neuroimaging study

**DOI:** 10.1192/j.eurpsy.2023.955

**Published:** 2023-07-19

**Authors:** K. Sim, Q. Chew, B. Prakash

**Affiliations:** 1 IMH/National Healthcare Group; 2 Institute of Bioengineering and Bioimaging, Agency for Science, Technology and Research, Singapore

## Abstract

**Introduction:**

The heterogeneity of schizophrenia (SCZ) regarding clinical features including symptomatology, disease course and their inter-relationships with underlying biological substrates remain incompletely understood.

**Objectives:**

In a bid to reduce illness heterogeneity using biological substrates, our study aimed to employ brain neurostructural measures for subtyping SCZ patients, and evaluate each subtype’s relationship with clinical features such as illness duration, psychotic psychopathology, and deficit status.

**Methods:**

We recruited 240 subjects (160 SCZ patients, 80 healthy controls) for this study. All participants underwent brain structural magnetic resonance imaging scans and clinical assessments using the Positive and Negative Syndrome Scale. Biological subtypes of SCZ were identified using “Heterogeneity through discriminative analysis” (HYDRA), a clustering technique which accounted for relevant covariates and the inter-group normalized percentage changes in brain volume were also calculated.

**Results:**

We found two neuroanatomical subtypes (SG-1 and SG-2) which were found amongst our patients with SCZ. The subtype SG-1 was associated with enlargements in the third and lateral ventricles, volume increase in the basal ganglia (putamen, caudate, pallidum), longer illness duration, and deficit status. The subtype SG-2 was associated with reductions of cortical and subcortical structures (hippocampus, thalamus, basal ganglia).

**Conclusions:**

These findings have clinical implications in the early intervention, response monitoring, and prognostication of SCZ. Future studies may adopt a multi-modal neuroimaging approach to enhance insights into the neurobiological composition of relevant subtypes.

**Disclosure of Interest:**

None Declared

